# The self–other knowledge asymmetry in cognitive intelligence, emotional intelligence, and creativity

**DOI:** 10.1016/j.heliyon.2018.e01061

**Published:** 2018-12-24

**Authors:** Aljoscha C. Neubauer, Anna Pribil, Alexandra Wallner, Gabriela Hofer

**Affiliations:** University of Graz, Institute of Psychology, Graz, Austria

**Keywords:** Psychology

## Abstract

The self–other knowledge asymmetry model (SOKA) assumes that some personality traits might be open to oneself and other persons (‘open area’), while other traits are more accurately perceived by others (‘blind spot’); a third group of traits might be visible only to oneself and not to others (‘hidden area’), and finally a trait might neither be visible to oneself nor to one's peers (‘unknown area’). So far, this model has been tested only for personality traits and general intelligence, not for more specific abilities; to do so was the novel intention of our study. We tested which of six abilities (verbal, numerical, and spatial intelligence; interpersonal and intrapersonal competence; and creative potential/divergent thinking ability) are in which SOKA area. We administered performance tests for the six abilities in two samples – 233 14-year-olds and 215 18-year-olds – and collected self- and peer-ratings for each domain. Numerical intelligence and creativity were judged validly both from self- and peer-perspectives (‘open area’). In the younger sample verbal intelligence was validly estimated only by peers (‘blind spot’), whereas the older group showed some insight into their own abilities as well (‘blind spot’ to ‘open area’). While in the younger group only the pupils themselves could validly estimate their intra- and interpersonal competence (‘hidden area’), in the older group peers were also successful in estimating other's interpersonal competence, albeit only with low accuracy (‘hidden area’ to ‘open area’). For 18-year-olds, spatial ability was in the hidden area too, but in 14-year-olds this could neither be validly estimated by pupils themselves nor by peers (‘unknown area’). These results implicate the possibility of non-optimal career choices of young people, and could, therefore, be helpful in guiding professional career counselling.

## 1 Introduction

“Why do others sometimes know things about us that we don't know about ourselves?” (Vazire, 2010, p. 281). This tricky question was asked by Simine Vazire, the author of the so-called self–other knowledge asymmetry model (SOKA), in which she described the phenomenon that many people have surprisingly insufficient insight into their own personality. This violates the common-sense conviction postulated by Augustin and Descartes that nobody knows yourself better than you do. Although self-ratings of personality can be a valid source when predicting professional and other life outcomes, and in spite of the fact that self-perceptions play a very important role in current conceptions of personality (Roberts et al., 2007), it has also repeatedly been shown that people have limited insight into their own traits (Beer and Vazire, 2017; Vazire and Mehl, 2008).

**Fig. 1:**
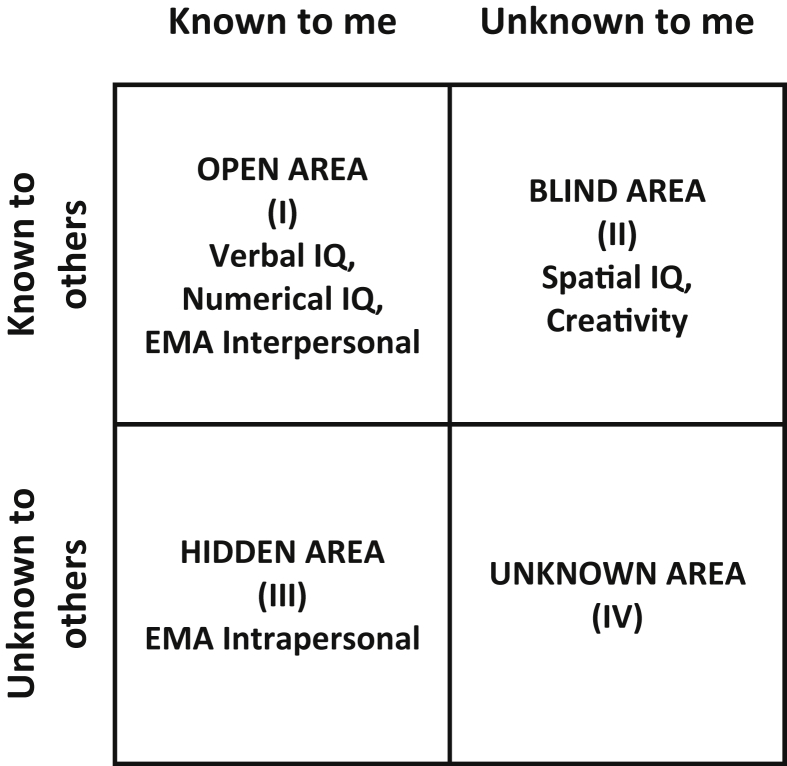
Adaptation of the Johari window (Luft and Ingham, 1955) and hypotheses about abilities. EMA = Emotional Management Ability.

Much research has shown compelling evidence for the validity of informant reports, such as peers or even unknown persons, called zero-acquaintance judgements (Borkenau and Liebler, 1992). That there does not have to be an overlap between self-judgements of personality and informant judgements has been demonstrated in studies (Carlson, 2013; Helzer and Dunning, 2012; Vazire and Carlson, 2011) inspired by Vazire's (2010) proposition of the SOKA model. She started her research on the basis of Luft and Ingham's Johari window (1955), which contains four quadrants (see Fig. 1): (a) aspects of personality known to both the self and others (open area); (b) aspects not known to the self but known to others (blind area); (c) aspects known to the self but not to others (hidden area); and (d) aspects unknown to both self and others (unknown).

As pointed out by Vazire (2010), the problem of people's limited insight into their personality traits not only has important theoretical implications, but also practical ones in several fields, including clinical psychology as well as in work and organizational psychology. So far, the SOKA model has been studied mostly in the field of classical personality traits like the Big Five, as well as in two very general abilities (general intelligence and creativity). In several studies (e.g., Vazire, 2010; see also Beer and Vazire, 2017; Bollich et al., 2011) these authors had participants providing self-ratings and informant ratings from friends and from strangers in a round-robin design. Through this approach, Vazire (2010) showed that in the open area (a) there are the observable, nonevaluative traits like extraversion; in the blind spot (b) there are evaluative traits like intelligence, and in the hidden area (c) there are internal (unobservable) non-evaluative traits like neuroticism.

Since then, surprisingly little research has taken up the general idea; the few studies on the SOKA model deal solely with clinical fields (Carlson et al., 2013) or social relations (Uziel, 2012).

What is completely unknown is: Where in the SOKA model are specific cognitive abilities like verbal, numerical and spatial intelligence, or other abilities like creativity and emotional competencies located? So far, no study has simultaneously assessed a broad variety of well-known abilities/performance traits and at the same time obtained self- as well as informant-ratings of the very same abilities. Therefore, we want to analyze the location of six defined abilities in the SOKA model: verbal, numerical and spatial intelligence (which are part of most current intelligence models; Hunt, 2010), creativity (or more exactly the creative potential as measured by divergent thinking tasks), as well as two central factors of emotional intelligence, namely intra- and interpersonal intelligence/competence (Freudenthaler and Neubauer, 2005).

This research question is not only relevant from a theoretical point of view but also has important practical implications regarding career counselling, especially for young people: Are they (or their peers, parents, etc.) aware of their abilities and talents, so that they can base decisions regarding their vocational choices not only on their interests but also on their abilities? This is particularly important, considering that abilities and interests mostly display surprisingly low relationships, as shown in the meta-analysis by Pässler et al. (2015). The large importance of abilities for later professional success has been documented in several meta-analyses (Schmidt and Hunter, 2004, 1998; Strenze, 2007), which mostly showed correlations around .5 for cognitive intelligence tests. For the big five, however, meta-analytically smaller correlations with job success have been reported, mostly between .1 and .2, maximally up to .3 (Barrick et al., 2001). For emotional abilities, however, evidence on the importance for job success is much less clear. Two recent meta-analyses (Andrei et al., 2016; Joseph, Jin, Newman, & O'boyle, 2015) demonstrated that also ‘emotional intelligence’ (EI) is relevant for job success, and although the relationships are small, EI shows an incremental contribution over and above cognitive intelligence. For creativity, however, no meta-analysis on relationship with job success is available.

What do we know about the validity of self- versus informant ratings of abilities? Except for Vazire's (2010; Vazire and Mehl, 2008) studies, so far no one has obtained performance measures, self- and informant ratings in the same study. To derive hypotheses, we thus needed to look into studies of (a) self-estimates of abilities and (b) informant estimates of abilities.

A recent metasynthesis by Zell and Krizan (2014) has shown that self-estimates of quite diverse abilities are of low to medium validity; the mean correlation being only .29, with a considerably higher average correlation only for verbal ability (*r* = .63), whereas for some abilities it was considerably lower than .29 – in particular, for nonverbal skills it was only .09. Another meta-analysis by Freund and Kasten (2012), which focused on abilities covered in intelligence tests, however, reported valid self-estimates only for numerical but not verbal and spatial abilities. This discrepancy might have arisen due to different conceptualizations of verbal abilities: In their meta-analysis, Freund and Kasten referred to verbal intelligence (as measured in intelligence tests), whereas the meta-synthesis by Zell and Krizan referred to foreign language ability.

With regard to informant estimates of abilities, two kinds of studies can be found: First, some studies analyzed the validity of zero-acquaintance reports for general intelligence, which is mostly around .3 (Borkenau and Liebler, 1993; Borkenau et al., 2004). Studies on peer reports of intelligence have been summarized by Denissen et al. (2011), who concluded that they “were reliable, stable and weakly correlated with objective intelligence” (p.108). The found intelligence*peer correlations that varied from .22 to .27.

A further restriction is that pertinent research has been conducted only in adults, not in children or adolescents. However, from the perspective of career counseling we argue that the validity of self- and informant estimates of abilities is particularly important for young people facing important career decisions. In choosing a career path, young people might either rely on the self-perception of their abilities, or seek advice from friends, parents, teachers, and so on. But do self-estimates of abilities and/or informant estimates of abilities give a sufficiently valid picture of the real (psychometrically determined) ability? And for which abilities should (young) people rely on their self-perception or on the advice of informants?

In order to answer these questions, we want to analyze validity of self- versus informant ratings of select abilities at two age points, where important career decisions are made. These are at 13/14 and at 18 years of age. In many Western countries adolescents around 13–14 years of age decide whether to choose a further career at (higher secondary) school leading eventually to academic studies at university, or whether to go directly into professional education (such as apprenticeships in Germany, Austria, other countries; see Dumfart et al., 2016).

The second timepoint for career decisions is at 17–18 years, when (senior) high school students decide about a career path at college or university. Therefore, we want to analyze the validity of self- and informant reports of select abilities at 14 compared to 18 years of age.

The following hypotheses are being tested:

1) Verbal and interpersonal ability should be located in the *open area* (I, see Fig. 1), that is, self- and informant reports should both be valid sources (Furnham et al., 2005; Vazire, 2010). We based our prediction regarding verbal ability on the meta-synthesis by Zell and Krizan (2014), instead of the meta-analysis by Freund and Kasten (2012), given the larger scale of the former. Our prediction for *inter*personal abilities arose from the finding that they usually correlate at medium-effect size with extraversion (Freudenthaler et al., 2008), which was in the open area in Vazire (2010).
2) For numerical ability, Freund and Kasten (2012) found valid self-estimates, whereas Zell and Krizan (2014) did not analyze this ability separately. With regard to informant ratings, no study was found that analyzed the validity of numerical ability. However, because of the high visibility of numerical ability in school contexts, we predict numerical ability to be also in the *open area* (I).
3) Spatial abilities have been shown not to be correlated with self-estimates (Freund and Kasten, 2012), therefore it could be in the *blind area* (II) where informant ratings of performance might be more valid. The same prediction is made for creativity (which was in the blind area in Vazire, 2010).
4) Finally, we expect *intra*personal competence to be located in the *hidden area* (III) of the SOKA window. Vazire (2010) placed neuroticism in the hidden area and in a former study we showed that intrapersonal competence is substantially correlated (negatively) with neuroticism (Freudenthaler and Neubauer, 2005).

Finally, we ran a set of more exploratory multiple regressions to see how good the six abilities can be predicted from self- and peer-perspective in combination. The aim was on one hand to see whether the self- and other perspective can complement each other and for which abilities they can do so. On the other hand, in case both perspectives could contribute, we wanted to see the total prediction (multiple *R*^2^) and compare that between the six abilities.

## 2 Method

### 2.1 Samples

We recruited two convenience samples of adolescents in lower secondary school (LSS; grade 8, around 13/14 years, *n* = 237) and higher secondary school (HSS; grade 12, around 17/18 years, *n* = 225). However, since we wanted to compare correlations between measures, we excluded all participants with missing values on any of our key variables (performance in ability tests, self-estimates, and peer estimates). This left 448 participants (55.8 % female), with 233 of them in LSS (*M*_age_ = 14.07, *SD*_age_ = 0.60, 53.2 % female) and 215 of them in HSS (*M*_age_ = 18.00, *SD*_age_ = 0.70, *n* = 215, 58.6 % female).

The studies were approved by the Ethic Commission of the University of Graz and the School council of the Austrian province of Styria. According to Austrian law, this fulfills the requirements for consent as long as tested pupils have the right to refuse – which they had in the studies conducted. We aimed at recruiting a diverse and representative sample, therefore inviting pupils from both public and from private schools that were located in rural areas and cities across two Austrian provinces (Styria and Upper Austria). A total number of thirteen schools participated.

### 2.2 Measures

Descriptive statistics and reliabilities of all measures can be found in Table 1.

**Table 1:** Descriptive statistics and reliabilities of performance measures, self-estimates, and peer estimates.

Ability	Performance			Self-estimate			Peer estimate		
	*M*	*SD*	Rel.	*M*	*SD*	Rel.	*M*	*SD*	Rel.
Lower secondary school									
Verbal	12.30	3.61	.61 [.54, .68]	3.26	0.69	.82 [.79, .86]	3.23	0.71	.93 [.91, .94]
Numerical	8.17	4.41	.86 [.84, .89]	3.25	0.90	.92 [.91, .94]	3.27	0.79	.96 [.96, .97]
Spatial	6.90	3.25	.66 [.60, .72]	3.43	0.73	.84 [.81, .87]	3.29	0.56	.90 [.88, .92]
Creativity	1.27	0.32	κ = .74	3.43	0.71	.82 [.78, .85]	3.28	0.60	.87 [.84, .89]
EMA intrapersonal	2.83	0.38	.49 [.38, .59]	3.36	0.59	.64 [.56, .71]	3.24	0.45	.74 [.69, .78]
EMA interpersonal	2.97	0.42	.58 [.49, .66]	3.65	0.67	.83 [.79, .86]	3.32	0.54	.85 [.82, .87]
Higher secondary school									
Verbal	17.29	3.30	.57 [.48, .65]	3.30	0.58	.80 [.75, .84]	3.44	0.65	.92 [.90, .94]
Numerical	10.74	4.24	.85 [.82, .87]	3.11	0.88	.93 [.91, .94]	3.34	0.76	.96 [.95, .97]
Spatial	9.78	2.75	.74 [.69, .79]	3.50	0.70	.85 [.82, .88]	3.55	0.46	.88 [.85, .90]
Creativity	1.53	0.32	κ = .74	3.36	0.66	.82 [.78, .85]	3.46	0.55	.86 [.83, .89]
EMA intrapersonal	2.96	0.36	.49 [.38, .58]	3.51	0.54	.63 [.55, .70]	3.50	0.50	.80 [.76, .84]
EMA interpersonal	3.08	0.38	.55 [.45, .63]	3.74	0.57	.79 [.75, .83]	3.53	0.57	.89 [.87, .91]

*Note.* EMA = Emotional management ability. Rel. = reliability, measured as (1) inter-rater agreement between both judges in form of Cohen's kappa (κ) for the performance measure of creativity or (2) Cronbach's alpha for all other variables. Values in brackets refer to 95% confidence intervals for Cronbach's alpha, calculated as proposed by Bonett and Wright (2015).

#### 2.2.1 Intelligence

Verbal, numerical and spatial abilities were measured by means of three subtests from Intelligenz-Struktur-Analyse (ISA; Fay et al., 2001). The subtests were for verbal ability ‘Gemeinsamkeiten finden’ (commonalities), numerical ability ‘Zahlenreihen’ (number series) and spatial ability ‘Figurenauswahl’ (figure completion), and these are well-established, reliable, and valid measures of these abilities for use in German language.

#### 2.2.2 Creativity

Creativity cannot be assessed directly, particularly not in these young people. Therefore, we measured the creative potential through divergent thinking tasks. We used three alternative uses tasks, namely umbrella, plastic bottle, and shoe, each given with a time limit of 2 min.; these tests have proven reliable and valid in a former study (Jauk et al., 2013). In these tasks, the students had to name as many original uses as they can for the given object within the time limit. Each task was analyzed for fluency (number of answers) and originality, the latter being judged independently by three raters (authors A.P. and A.W. and a student; see Table 1 for interrater-agreement measure κ). Since the results for both measures showed similar tendencies but those for fluency were less clear-cut, we only report the originality measure here (see also Diedrich et al., 2018). Fluency and originality were correlated at .53 in LSS and at .60 (both *p* <. 001) in HSS.

#### 2.2.3 Emotional abilities/competencies

Emotional abilities were measured by means of a previously developed test for emotional management ability (EMA, inter- and intrapersonal) for which reliability (αs > .7) and validity has been demonstrated (Freudenthaler et al., 2006; see also Freudenthaler and Neubauer, 2005; Freudenthaler et al., 2008). This is a 28-item situational judgement test (SJT) with 14 items each for intrapersonal and interpersonal emotional management ability. Items are of the following type: A short description of an emotionally laden situation is given and the testee has to choose from four response alternatives, which are scored between one and four where ‘4’ = maximally efficient handling of the situation and ‘1’ = least efficient handling of the respective situation. In the present study alphas were rather low (see Table 1), which is surprising considering that it was acceptable in prior studies. However, this is a common phenomenon with tests for social and emotional abilities (see Archer and Akert, 1977; Costanzo and Archer, 1989).

### 2.3 Self- and peer estimates

For each of the six performance domains a self- and a corresponding peer-estimate scale was developed by describing the respective ability in 9–10 items, reflecting its various aspects as good as possible. Peer estimates came from classmates: Each target person was rated by two peers/classmates, who were selected randomly. For each ability, the two peer-estimates were averaged, in order to obtain a measure that is less susceptible to distortions by extreme values. Items for the self-estimate questionnaires were statements like “I can easily rephrase a text using different wording.” (verbal intelligence) or “Estimating the size of a room is easy for me.” (spatial intelligence). The peer estimate questionnaires were based on the same items but slightly rephrased so that they referred to others instead of oneself (e.g., “_____ can easily rephrase a text using different wording.”) Participants were instructed to mentally fill the blanks with the names of the peers they were to judge. The appendix shows translations of all items used. As can be seen in Table 1, all alphas were either high (α > .80) or at least satisfactory (α > .70), with the exception of the self-estimate for intrapersonal ability (α_LSS_ = .64, 95% CI [.56, .71], α_HSS_ = .63, 95% CI [.55, .70]).

### 2.4 Procedure

Tests were given in regular class hours and took two hours and 50 minutes each. The order of tests was: 1. verbal, numerical and figural tests with time limits of 8, 11 and 7 minutes, respectively; 2. the three alternative uses tasks with a time limit of 2 minutes each; 3. the test for emotional management ability without a strict time limit (this took about 20 minutes). In order to obtain a maximum estimate of self*ability correlation, each self-estimate was given directly after the corresponding performance test. Peer estimates were taken at the end of each testing session, that is, after all other tests had been administered. At this point, individuals also had to complete the German version of the Inclusion of the Other in the Self Scale (IOS scale; Aron et al., 1992) for each peer they rated. This is a measure of relationship closeness and resulted in a score between one (no relationship at all) and seven (very close relationship/best friends).^1^

### 2.5 Statistical analyses

The validity of self- and peer estimates of abilities was investigated in two ways: First, we computed correlations between self-estimates and performance, as well as, between peer estimates and performance for each of the six ability domains. Bonferroni-correction was applied to all tests of significance involving multiple subscales of the same test, that is the ISA with three subscales (*p* = .05/3; resulting in a significance threshold of *p* < .0167), and the EMA with two subscales (*p* = .05/2; resulting in a significance threshold of *p* < .025). In addition to this significance-testing approach, we also developed a more descriptive approach when considering the minimal effect necessary to define a perspective as reasonably accurate. The aim of this effect-size-based approach is to obtain results that are comparable across studies and less dependent on sample size than those obtained by looking at significance levels alone. Similar to Vazire and Mehl (2008), we consider all estimate*performance correlations starting from *r* = .2 to indicate a relevant level of accuracy. This lies exactly between what would be considered a small and a medium sized effect according to common conventions and appears to be a reasonable threshold, considering the associations between self- or peer estimates and abilities found in past studies (Denissen et al., 2011; Freund and Kasten, 2012; Zell and Krizan, 2014). We then compared the resulting self-estimate*performance and peer estimate*performance correlations with each other. In line with Vazire (2010), we defined a difference between two correlation coefficients of more than .15 as substantial.

Second and with respect to the initially proposed practical research question of whether self- and informant views of one's own abilities could provide independent contributions to predicting actual (psychometric) performance, we performed a series of multiple regression analyses with performance in each of the ability tests as outcome. Gender was entered in the first step as control variable. In the second step, self- and peer estimates of the respective ability were added simultaneously to assess how much unique information each perspective can provide. All common assumptions of multiple regressions (i.e., homoscedasticity, absence of multicollinearity, independent errors, and normality of residuals) were assessed and met.

## 3 Results

Intercorrelations of all performance measures for both samples are displayed in Table 2. As can be seen, all classical intelligence facets (i.e., verbal, numerical, and spatial intelligence) correlated positively with each other in both samples, as did both facets of emotional management ability. While creativity correlated with all classical intelligence facets in the older sample, it correlated with verbal and numerical but not with spatial intelligence in the younger sample. There was no significant difference between the closeness of the friendship between the first peer and the target (LSS: *M* = 3.31, *SD* = 2.14; HSS: *M* = 3.13, *SD* = 1.72) and the one between the second peer and the target (LSS: *M* = 3.36, *SD* = 2.11, *t*(224) = −0.22, *p* = .826, *d* = 0.02; HSS: *M* = 3.37, *SD* = 1.83, *t*(212) = −1.44, *p* = .153, *d* = 0.10).

**Table 2:** Summary of intercorrelations of all performance measures as a function of school type.

Measure	1	2	3	4	5	6
Verbal (1)	–	.366**	.408**	.262**	−.028	.022
Numerical (2)	.451**	–	.371**	.143*	.060	−.122
Spatial (3)	.354**	.425**	–	.299**	.027	−.042
Creativity (4)	.295**	.281**	.122	–	.097	.075
EMA intrapersonal (5)	−.012	.130*	−.067	−.085	–	.262**
EMA interpersonal (6)	.101	.062	−.004	.123	.338**	–

*Note*. EMA = Emotional Management Ability. Intercorrelations for pupils of higher secondary schools (*n* = 215) are presented above the diagonal, and intercorrelations for pupils of lower secondary schools (*n* = 233) are presented below the diagonal. *p < .05. **p < .01.

### 3.1 Correlative findings

Table 3 shows the correlations of the six performance domains with self- and peer estimates for the respective domain in lower versus higher secondary school, as well as the self*peer correlations. Relationships of abilities with self- and peer estimates are quite diverse. Surprisingly, verbal intelligence does not correlate with self-estimates in lower secondary school. It also shows only a small to medium correlation with self-estimates in higher secondary school and, albeit significant, this correlation is slightly below the above defined accuracy threshold of *r* ≥ .2. Even peer estimates of verbal intelligence are only of rather low validity (*r*s between .26 and .28, albeit significant). Considering Vazire's (2010) suggestions that a difference in *r* values of more than .15 indicates a relevant difference between the perspectives, we conclude that in the younger sample the peer-perspective is somewhat more valid than the self-perspective, while in the older sample the difference falls short of that criterion. In contrast, numerical abilities show mostly medium to high validities, both for self- and peer estimates. Nevertheless, in higher secondary school the self-perspective is substantially more valid than the peer-perspective (difference in *r*s of .18). Spatial ability is quite badly self- and peer estimated in both samples, with the exception of self-estimates in the older age group; only in this group the difference between both perspectives is .15. Creativity (originality in divergent thinking tasks) gave low to medium validities (*r*s between.22 and .34); and according to the ‘Vazire criterion’ the differences in perspectives are irrelevant. Intrapersonal ability can be self-estimated well, with medium to high *r* values (*r*s between .37 and .39), whereas peers show no insight into this ability; the difference in *r* values exceeds .15 in both samples. For interpersonal abilities self-estimates show good validity (*r*s around .46), whereas peer-estimates show low validity in the older sample (*r* = .19, significant but below our proposed threshold) and no correlation with performance in the younger sample (*r* = −.01). Also here the difference between perspectives is substantial and conforms to the ‘Vazire criterion.’

**Table 3:** Correlations of abilities (performance) with self- and peer-estimates and correlations between self- and peer estimates for six abilities.

Ability	Lower secondary school			Higher secondary school		
	Perf * SE	Perf * PE	SE * PE	Perf * SE	Perf * PE	SE * PE
Verbal	.080 (*p* = .224)	.262* (*p* < .001)	.319* (*p* < .001)	.192* (*p* = .005)	.278* (*p* < .001)	.481* (*p* < .001)
Numerical	.427* (*p* < .001)	.455* (*p* < .001)	.446* (*p* < .001)	.533* (*p* < .001)	.357* (*p* < .001)	.472* (*p* < .001)
Spatial	.097 (*p* = .139)	.138 (*p* = .036)	.083 (*p* = .206)	.310* (*p* < .001)	.159 (*p* = .020)	.130 (*p* = .056)
Creativity	.335* (*p* < .001)	.217* (*p* < .001)	.219* (*p* = .001)	.315* (*p* < .001)	.278* (*p* < .001)	.315* (*p* < .001)
EMA intrapersonal	.374* (*p* < .001)	.019 (*p* = .769)	.044 (*p* = .500)	.388* (*p* < .001)	.130 (*p* = .057)	.089 (*p* = .192)
EMA interpersonal	.459* (*p* < .001)	−.009 (*p* = .894)	.037 (*p* = .576)	.476* (*p* < .001)	.190* (*p* = .005)	.280* (*p* < .001)

*Note.* EMA = Emotional Management Ability. Perf = Performance. SE = Self-Estimates. PE = Peer-Estimates. *significant (for tests with multiple subtests after Bonferroni correction; *p* < .0167 for analyses involving verbal, numerical or spatial intelligence; *p* < .025 for analyses involving EMA intra- or interpersonal).

Table 3 also shows self*peer correlations: In both samples self- and peer perspectives agree at medium to high size for verbal and numerical abilities, and (lower) for creativity. In higher secondary school there is a medium self*peer correlation for interpersonal ability. Spatial and intrapersonal abilities show (almost) no correspondence between self and peers.

### 3.2 Multiple regressions

The amount of variance explained in each step of the hierarchical multiple regression analyses as well as beta-values of each predictor can be seen in Table 4. Our control variable gender was a significant and positive predictor of numerical intelligences in both samples (albeit in LSS only in the first step), of spatial intelligence in lower secondary school and intrapersonal abilities in higher secondary school, indicating that boys performed better than girls in the respective performance measures. Girls outperformed boys in creativity in lower secondary school and interpersonal abilities in both age groups.

**Table 4:** Hierarchical multiple regression analyses predicting level of verbal, numerical, and spatial intelligence, creativity, and intra- and interpersonal emotional management abilities from corresponding self- and peer estimates.

Predictor	Ability											
	Verbal		Numerical		Spatial		Creativity		EMA intra-personal		EMA inter-personal	
	Δ*R*^2^	β	Δ*R*^2^	β	Δ*R*^2^	β	Δ*R*^2^	β	Δ*R*^2^	β	Δ*R*^2^	β
Lower secondary school												
Step 1	<.001		.043**		.043**		.022*		.003		.082**	
Gender		−.022		.208**		.207**		−.150*		.054		−.287**
Step 2	.070**		.231**		.015		.130**		.139**		.173**	
Gender		.034		.076		.187**		−.134*		.038		−.219**
SE		−.005		.249**		.032		.308**		.372**		.419**
PE		.273**		.335**		.117		.131*		.010		−.061
Total *R*^2^	.071**		.274**		.058**		.152**		.142**		.255*	
Higher secondary school												
Step 1	<.001		.091**		.009		<.001		.039**		.059**	
Gender		−.006		.302**		.096		−.013		.198**		−.243**
Step 2	.084**		.264**		.101**		.138**		.144**		.218**	
Gender		.046		.245**		.019		.066		.158*		−.219**
SE		.070		.407**		.290**		.251**		.353**		.459**
PE		.254**		.179**		.118		.217**		.118		.026
Total *R*^2^	.084**		.355**		.110**		.139**		.184**		.276**	

*Note.* EMA = Emotional Management Ability; SE = Self-Estimates; PE = Peer-Estimates. One participant in lower secondary school did not provide their gender, leading to a sample of *n* = 232 for all regression analyses involving this sample. Gender was scored such that positive betas indicate higher ability levels in men than in women. *p < .05. **p < .01.

In both samples, verbal intelligence can only be predicted from peer estimates and the overall amount of variance explained is comparatively low (LSS: *F*(3, 228) = 5.80, *p* = .001, *R*^2^ = .071, Cohen's *f*^2^ = .076; HSS: *F*(3, 211) = 6.42, *p* < .001, *R*^2^ = .084, Cohen's *f*^2^ = .092). Numerical intelligence can be predicted with high effect sizes and both self- and peer estimates contribute significantly to its prediction (LSS: *F*(3, 228) = 28.67, *p* < .001, *R*^2^ = .274, Cohen's *f*^2^ = .377; HSS: *F*(3, 211) = 38.69, *p* < .001, *R*^2^ = .355, Cohen's *f*^2^ = .550). Spatial intelligence shows low predictability in both samples and can only be predicted from self-estimates in higher secondary school, while neither self- nor peer estimates can contribute to its prediction in lower secondary school (LSS: *F*(3, 228) = 4.66, *p* = .003, *R*^2^ = .058, Cohen's *f*^2^ = .062; HSS: *F*(3, 211) = 8.73, *p* < .001, *R*^2^ = .110, Cohen's *f*^2^ = .124). For creativity, a medium-sized amount of variance can be explained and both perspectives contribute to its prediction (LSS: *F*(3, 228) = 13.62, *p* < .001, *R*^2^ = .152, Cohen's *f*^2^ = .179; HSS: *F*(3, 211) = 11.32, *p* < .001, *R*^2^ = .139, Cohen's *f*^2^ = .161). When it comes to intrapersonal emotional management abilities, only self-estimates contribute to its prediction and the overall amount of explained variance is also medium-sized (LSS: *F*(3, 228) = 12.53, *p* < .001, *R*^2^ = .142, Cohen's *f*^2^ = .166; HSS: *F*(3, 211) = 15.81, *p* < .001, *R*^2^ = .184, Cohen's *f*^2^ = .225). Like numerical intelligence, interpersonal emotional management abilities can be predicted with high effect sizes in both samples. However, here only self-estimates can significantly contribute to the prediction (LSS: *F*(3, 228) = 26.03, *p* < .001, *R*^2^ = .255, Cohen's *f*^2^ = .342; HSS: *F*(3, 211) = 26.87, *p* < .001, *R*^2^ = .276, Cohen's *f*^2^ = .381).

## 4 Discussion

The questions and hypotheses of this study were derived from three sources: 1. Vazire's (2010) SOKA model; 2. the literature on self-estimates of abilities; 3. the validity of informant ratings of abilities. We made the following predictions regarding the four quadrants of the SOKA window (see Fig. 1): Verbal and numerical, as well as interpersonal ability should be in the open quadrant (I); spatial intelligence and creativity in the blind spot (II); intrapersonal ability in the hidden area (III).

Most predictions were not confirmed. Since the pattern of results was similar for both age groups, we discuss most findings without differentiating between age groups (unless otherwise noted):

In lower secondary school verbal intelligence was not located in the open area but in the blind spot, meaning that these adolescents have no accurate self-views here, while peers judge them more accurately, but also with rather low validity (*r* of .26). Also in line with this result, only peer estimates could significantly predict verbal intelligence in the respective multiple regression analysis. Pupils in higher secondary school seem to have at least some sense of their verbal abilities, at least according to classical significance-testing. However, the correlation is slightly below our threshold of .2, speaking for low validity and putting verbal intelligence at the verge between the open and blind spot. Again, peers have a bit more insight, albeit the difference between both perspectives is lower than what Vazire (2010) suggested to be relevant. The accuracy of both perspectives was on the one hand unexpectedly low, considering the supposedly high visibility of this ability and the findings on language competence from Zell and Krizan's metasynthesis (2014). On the other hand, it conforms well with results from Freund and Kasten's meta-analysis (2012), which operationalized verbal abilities as performance in verbal intelligence subtests and could therefore be considered more alike to the present study. Moreover, multiple regression showed that verbal ability had (among) the lowest total ‘estimability’ (*R*^2^s of .07 and .08), putting it almost in the unknown area. This might be the most puzzling finding of the current study.

There are several possible explanations for the low associations between psychometrically measured and estimated verbal intelligence: The verbal intelligence test might not be a good measure of verbal ability, but the ISA used here is a well-established verbal intelligence test suited for people from 14 years and older, which has satisfactory reliability and also showed the expected intercorrelations with the other intelligence factors (see Table 2). In addition, the substantial correlations with divergent thinking tasks clearly speak in favor of a valid measurement of verbal ability in our samples (verbal fluency, which is also measured in divergent thinking tasks, is an important aspect of verbal ability, see Thurstone and Thurstone, 1943). The self- and the peer-estimate scales for verbal ability were of sufficient reliability (see as in Table 1) and the self-peer correlations were rather high (see Table 3). Therefore, it seems rather that the implicit concept of verbal ability in adolescents might not correspond to our (the psychologists) concept of verbal intelligence. A closer look at the items in the self- and peer estimate scales shows that some items might reflect oral expression abilities, which might not be correlated substantially with the abilities tapped by verbal intelligence tests.

There is another – maybe most plausible – explanation though: These days, the supposedly weak verbal proficiency of young people is often complained about, with the low rate of (focused) reading and other factors being held responsible (e.g., De Naeghel, Van Keer, Vansteenkiste and Rosseel, 2012). This might be a well-founded reason, with the following argumentation: Individuals of lower verbal ability might be particularly weak at self- and peer estimating because this might require exactly what they are missing: a good verbal ability (see also the Dunning-Kruger-effect; Kruger and Dunning, 1999; Dunning et al., 2003).

The finding that numerical ability is in the open area is in agreement with Freund and Kasten's meta-analysis (2012) and can presumably also be explained by the high visibility of this ability in the context of school classes (i.e., adolescents' mathematical performance) and the amount of feedback pupils get in this context.

Spatial intelligence we had hypothesized to be located in the blind spot. However, it turned out to be in the unknown area (14 years) or in the hidden area (18 years). This is the only ability that changes its position within the Johari window between both age groups. This seems less surprising than some other findings, as spatial abilities might not play a major role in school performance and therefore do not receive as much feedback from school performance in the younger sample compared to the higher secondary school, where in math classes aspects of spatial ability are also required (e.g., geometry).

The third unexpected finding is that creativity is not in the blind spot (Vazire, 2010) but in the open area. We also found that self-ratings of creativity were of medium validity, being even higher than those from the informant perspective. This deviation from Vazire's (2010) finding might be explained by the age group studied here. Children/adolescents might get more feedback in school about their creativity than adults usually get, but this is only a conjecture.

Apart from verbal intelligence, maybe the second most surprising result is that, especially in the younger age group, interpersonal competence was not located in the open area but in the hidden area. This means, young adolescents have accurate self-views but no insight into each other's interpersonal abilities. Here it might be that – after filling out the SJT for interpersonal ability – the testees have a conceptually valid view of their own interpersonal competence. However, when judging peers (which was done at the end of class) that conception might have vanished already. It is also possible that the peer estimate reflects some general aspects related to liking, although this is purely speculative at the moment. Nevertheless, it is interesting to note that this ability is the only one where some change in peers' accuracy with age can be seen: While in 14-year-olds peer estimates showed zero validity, in the 18-year-olds it was around .19 and even though this is still slightly below our threshold for accuracy, the change between both age groups is considerable. Self-peer-agreement also rose from .04 to .28. This means that interpersonal competence might need some ontogenetic development – and possibly also feedback from experiences with the other gender – so that people can properly estimate other peoples' emotional competence. In explaining the weak validity of peer estimates, one could also be tempted to blame the performance test itself. However, upon closer examination the test mostly showed the expected correlations with other abilities, namely medium-size correlations of .34 in the younger sample and .26 in the older sample with intrapersonal ability (see Table 2), which corresponds well with earlier findings (Freudenthaler and Neubauer, 2005; Freudenthaler et al., 2008).

Finally, the fact that intrapersonal competence is in the hidden area conforms well with the initial prediction.

Turning to the practical issue of career counseling in the field of work and organizational psychology, using multiple regression analyses we asked whether complementing self- and peer perspectives of six central ability domains gives the adolescents a good perspective on their real performance level in the respective domains. Numerical ability as well as interpersonal ability can be judged quite well in terms of the amount of variance explained (≥25%). In the case of numerical ability both the self- and the peer-perspective make important contributions to its prediction, while for interpersonal ability only the self-perspective can contribute (Table 4). On the contrary, spatial and verbal ability have quite low levels of explained variance (≤11%), implicating that young people who face the situation of selecting a career have no accurate account of either their verbal or their spatial abilities. Intrapersonal abilities and creativity lie in between with around 15% variance explained.

These findings have important practical implications (see also Zell and Krizan, 2014): Spatial ability in particular appears to be a construct, for which neither oneself nor others can make an accurate judgement of the real performance level. This is not surprising – we usually do not get much feedback on our spatial ability, particularly not at a very young age. Somehow more surprising is the low validity of self- and peer perspectives on verbal ability, which might pose a serious problem for young people. Even though a replication of these findings is needed, it seems that in the present sample it is, if anything, the peer perspective that gave a satisfying although not high validity of verbal ability. It seems most puzzling how poor the self-estimates of verbal ability are, which could not be expected on the basis of Zell and Krizan's (2014) metasynthesis, but it is in line with Freund and Kasten (2012), as explained above. Thus, it seems that the weak self-estimation of verbal ability is a ubiquitous phenomenon and that the young people of our sample indeed have a weak meta-perception of their verbal proficiency.

Finally, between the well estimable numerical intelligence and interpersonal competence on one hand and the poorly estimable verbal and spatial intelligence on the other hand, we find intrapersonal ability and creativity. Between around 15 and 20 % of variance of these abilities can be estimated, which means (vocational) counselling is important here too, as people themselves as well as peers do not give the adolescents a particularly adequate estimate of how good they are in these domains.

The study does have some limitations: While our samples are rather large and we have put effort into making them representative for the specific age groups, it has yet to be tested whether the findings would generalize to (older) adults. We restricted our study to 13/14-year- and 18-year-olds here, because in these age ranges important decisions regarding vocational choices are being made (at least in countries with an apprentice system). A second restriction concerns the rather low reliability of both scales of the test for emotional management abilities and the self-estimates of intrapersonal abilities, which could have potentially impacted the correlations in these domains. As already stated, this is not uncommon with tests for social and emotional abilities (Archer and Akert, 1977; Costanzo and Archer, 1989). Bonett and Wright (2015) argued that Cronbach's alpha often underestimates internal consistency and suggested always reporting the confidence interval around it. As can be seen in Table 1, upper limits for Cronbach's alpha are around what is considered acceptable for self-estimated intrapersonal skills but still potentially problematic for the EMA performance measures. Nevertheless, self*performance correlations for inter- and intrapersonal ability were around .40 for both samples, which is why we do not expect a strong restriction of generalizability here. Moreover, as we gave self-estimates directly after each corresponding ability test, we think that we obtained rather ‘upper-limit’ estimates of the validities of the self-estimates. If the self- and also the peer-estimate scale were given out of the context of the ability testing, validities might have been lower. Another restriction might be that we did not take self-efficacy into account; this should be properly addressed in future studies.

Bearing in mind that Vazire (2010) proposed that acquaintance or relationship closeness between target and rater might be important for a trait's position within the SOKA model, future research should investigate their impact in a more systematic manner than the present study. For example, one could look into different groups of peer-raters with different kinds of relationships to the target. We have found no moderating influence of self-reported closeness of the friendship between peer-rater and target on the association between peer-ratings and performance. However, this result might have been affected by specifics of our research: We assigned peer-raters randomly, thereby reducing the possibility that raters were the participants' best friends or classmates who do not like them at all. Since relationship closeness was not our main interest in this study, we even further minimized its potential implications by using the average of both peer-raters in our analyses.

Similarly, it might be interesting to investigate gender differences when it comes to the accuracy self- and peer estimates of intelligence. Some research indicates that men give higher self-estimates of intelligence than women do (see the meta-analysis by Syzmanowicz and Furnham, 2011) and that others also give higher intelligence-estimates for men than for women (Borkenau et al., 2004; Steinmayr and Spinath, 2009), both being particularly apparent for spatial and numerical intelligence. There is also some indication that these higher self- and other-estimates do not reflect existing gender differences in actual intelligence alone, but also a tendency for overestimation (Steinmayr and Spinath, 2009; Syzmanowicz and Furnham, 2011). We found gender differences in intelligence and emotional competence that are largely consistent with existing literature (Freudenthaler et al., 2008; Halpern & LaMay, 2000). Therefore, it would be interesting to see whether gender also affects the position of an ability within the SOKA model. However, the aim of the present study was to provide first insights the allocation of abilities into the model, which is why we controlled for gender in our regression analyses instead of investigating it as potential moderator.

Summarizing, and viewed from a practical career counseling perspective, the weak self- and peer validities corroborate quests for professional career counseling on the basis of psychometrically sound ability tests. As young people might have biased views of their own but also of their peers' abilities, scientifically based career counseling should include not only tests for interests but also performance tests to avoid erroneous vocational decisions.

## Declarations

### Author contribution statement

Aljoscha Neubauer: Conceived and designed the experiments; Analyzed and interpreted the data; Wrote the paper.

Anna Pribil, Alexandra Wallner: Performed the experiments; Analyzed and interpreted the data.

Gabriela Hofer: Analyzed and interpreted the data; Contributed reagents, materials, analysis tools or data.

### Funding statement

This research did not receive any specific grant from funding agencies in the public, commercial, or not-for-profit sectors.

### Competing interest statement

The authors declare no conflict of interest.

### Additional information

Data associated with this study has been deposited at https://doi.org/10.17605/OSF.IO/V8E5X.

## Footnotes

1 According to Vazire (2010), acquaintance or relationship closeness might affect the accuracy of peer-estimates. Therefore, we ran a set of multiple regression analyses with the ability estimate of each peer as predictor, their self-reported closeness of the friendship to the target as moderator, and the performance measure of the respective ability as outcome. Since these analyses resulted in no major influence of relationship closeness and were not central to our hypotheses, we refrain from reporting them in more detail.
