# Supplementary material for: The self–other knowledge asymmetry in cognitive intelligence, emotional intelligence, and creativity
Source: Heliyon. 2018 Dec 24;4(12):e01061. doi: 10.1016/j.heliyon.2018.e01061 (PMC6307038; doi:10.1016/j.heliyon.2018.e01061)
Supplement: Appendix [file mmc1.docx]

Appendix

Self-Estimate Questionnaire 1

In the following section you will find a number of statements. Please answer honestly in the way that you think best describes your own opinion. Please answer this survey in a timely manner and bear in mind that it is only about your personal opinion. Please only mark one option per statement. There are no right or wrong answers to this.

|  |  | Strongly Disagree | Disagree | Uncertain | Agree | Strongly Agree |
| --- | --- | --- | --- | --- | --- | --- |
|  | Compared to other adolescents I have a very broad vocabulary. | O | O | O | O | O |
|  | I am good at expressing my thoughts and opinions verbally. | O | O | O | O | O |
|  | I read a lot and prefer sophisticated literature. | O | O | O | O | O |
|  | It is easy for me to find a synonym for a word. | O | O | O | O | O |
|  | I am very good at working out the meaning of an unknown word based on its context. | O | O | O | O | O |
|  | Making narrations more colourful is easy for me. | O | O | O | O | O |
|  | It is very easy for me to find as many words with the same initial letter as possible. | O | O | O | O | O |
|  | I can easily rephrase a text using different wording. | O | O | O | O | O |
|  | Reading difficult texts and understanding their meaning is a simple task for me. | O | O | O | O | O |
|  | I am extremely talented in language. | O | O | O | O | O |

|  |  | Strongly Disagree | Disagree | Uncertain | Agree | Strongly Agree |
| --- | --- | --- | --- | --- | --- | --- |
|  | Solving extremely complicated arithmetic problems is very easy for me. | O | O | O | O | O |
|  | I am good at interacting with numbers and operators. | O | O | O | O | O |
|  | It is easy for me to solve mathematical tasks and exercises. | O | O | O | O | O |
|  | Recognizing logical rules is a simple task for me. | O | O | O | O | O |
|  | I have no problems adding the prices of different goods. | O | O | O | O | O |
|  | I have a very good understanding of numbers and find handling them easy. | O | O | O | O | O |
|  | I have good mental arithmetic skills. | O | O | O | O | O |
|  | I am good at reading and understanding written maths problems. | O | O | O | O | O |
|  | I am extremely talented in logical mathematical thinking. | O | O | O | O | O |
|  |  | **Strongly Disagree** | **Disagree** | **Uncertain** | **Agree** | **Strongly Agree** |
|  | When somebody describes their house to me I am very good at imagining what it looks like. | O | O | O | O | O |
|  | I find orientation in a foreign city using a map extremely easy. | O | O | O | O | O |
|  | I can very easily imagine what an object would look like from a different point of view. | O | O | O | O | O |
|  | I am good at explaining to other people which route to use. | O | O | O | O | O |
|  | I am good at finding my way in an unknown area. | O | O | O | O | O |
|  | Estimating the size of a room is easy for me. | O | O | O | O | O |
|  | I can easily imagine three-dimensional objects. | O | O | O | O | O |
|  | I find it easy to judge the distance between two points/locations I am extremely talented at spatial thinking. | O | O | O | O | O |
|  | I am extremely talented at spatial thinking. | O | O | O | O | O |

Self-Estimate Questionnaire 2

In the following section you will find a number of statements. Please answer honestly in the way that you think best describes your own opinion. Please answer this survey in a timely manner and bear in mind that it is only about your personal opinion. Please only mark one option per statement. There are no right or wrong answers to this.

|  |  | Strongly Disagree | Disagree | Uncertain | Agree | Strongly Agree |
| --- | --- | --- | --- | --- | --- | --- |
|  | I am good at finding original headlines for drawings or writings. | O | O | O | O | O |
|  | I constantly have new ideas. | O | O | O | O | O |
|  | In my spare time I like to be active in an artistic way. | O | O | O | O | O |
|  | I have the skill to look at a problem from different perspectives. | O | O | O | O | O |
|  | It is very easy for me to find different possible solutions to a problem. | O | O | O | O | O |
|  | I have quite unconventional and flamboyant ideas. | O | O | O | O | O |
|  | I am very curious and interested in the world. | O | O | O | O | O |
|  | I can find many different uses for everyday objects. | O | O | O | O | O |
|  | I am extremely talented at creative tasks. | O | O | O | O | O |

Self-Estimate Questionnaire 3

In the following section you will find a number of statements. Please answer honestly in the way that you think best describes your own opinion. Please answer this survey in a timely manner and bear in mind that it is only about your personal opinion. Please only mark one option per statement. There are no right or wrong answers to this.

|  |  | Strongly Disagree | Disagree | Uncertain | Agree | Strongly Agree |
| --- | --- | --- | --- | --- | --- | --- |
|  | If something really bothers me I usually try to understand why I feel like that. | O | O | O | O | O |
|  | If a situation really concerns me I try to avoid similar situations in the future. | O | O | O | O | O |
|  | If a topic really aggravates me I still try to stay calm in dealing with it. | O | O | O | O | O |
|  | I am very good at expressing my own feelings in a clear and understandable way. | O | O | O | O | O |
|  | If I am feeling down I usually know how to cheer myself up.’. | O | O | O | O | O |
|  | Even if others tell me I really did very well at something I am still unhappy with it. | O | O | O | O | O |
|  | I am very good at recognizing and differentiating my own feelings. | O | O | O | O | O |
|  | If necessary I can easily control my own feelings. | O | O | O | O | O |
|  | I am extremely talented at regulating my own feelings. | O | O | O | O | O |

|  |  | Strongly Disagree | Disagree | Uncertain | Agree | Strongly Agree |
| --- | --- | --- | --- | --- | --- | --- |
|  | I am very good at helping others feel better when they are feeling down – without simply distracting them from their problem. | O | O | O | O | O |
|  | My feelings tend to make me react in an inappropriate way to others. | O | O | O | O | O |
|  | If a friend is sad, I try to support them in finding a solution to the problem. | O | O | O | O | O |
|  | If I am struggling to find a solution to a friend’s problems I try to find somebody else who can offer them better help. | O | O | O | O | O |
|  | If I hurt somebody’s feelings I realize this and apologize. | O | O | O | O | O |
|  | I am good at empathizing. | O | O | O | O | O |
|  | I have lots of ideas for solving interpersonal problems. | O | O | O | O | O |
|  | When interacting with others I am very polite. | O | O | O | O | O |
|  | I am good at understanding the emotions of others and reacting to them in an appropriate way. | O | O | O | O | O |
|  | I am extremely talented at interacting with other people. | O | O | O | O | O |

Always mentally fill the _____ with a name:

Peer-Estimate Questionnaire 1

In the following section you will find a number of statements. Please answer honestly in the way that you think best describes _________’s opinion. Please answer this survey in a timely manner and bear in mind that it is only about ________’s personal opinion. Please only mark one option per statement. There are no right or wrong answers to this.

|  |  | Strongly Disagree | Disagree | Uncertain | Agree | Strongly Agree |
| --- | --- | --- | --- | --- | --- | --- |
|  | In comparison to other adolescents _______ has a very broad vocabulary. | O | O | O | O | O |
|  | _______ is good at expressing his/her thoughts and opinions verbally. | O | O | O | O | O |
|  | _______ reads a lot and prefers sophisticated literature. | O | O | O | O | O |
|  | It is easy for _______to find a synonym for a word. | O | O | O | O | O |
|  | _______ is very good at working out the meaning of an unknown word based on its context. | O | O | O | O | O |
|  | Making narrations more colourful is easy for _______. | O | O | O | O | O |
|  | It is very easy for _______to find as many words with the same initial letter as possible. | O | O | O | O | O |
|  | _______ can easily rephrase a text using different wording. | O | O | O | O | O |
|  | Reading difficult texts and understanding their meaning is a simple task for _______. | O | O | O | O | O |
|  | _______ is extremely talented in language. | O | O | O | O | O |

|  |  | Strongly Disagree | Disagree | Uncertain | Agree | Strongly Agree |
| --- | --- | --- | --- | --- | --- | --- |
|  | Solving extremely complicated arithmetic problems is very easy for _______ | O | O | O | O | O |
|  | _______ is good at interacting with numbers and operators. | O | O | O | O | O |
|  | It is easy for _______to solve mathematical tasks and exercises. | O | O | O | O | O |
|  | Recognizing logical rules is a simple task for _______. | O | O | O | O | O |
|  | _______ has no problems adding the prices of different goods. | O | O | O | O | O |
|  | _______ has a very good understanding of numbers and finds handling them easy _______. | O | O | O | O | O |
|  | _______ has good mental arithmetic skills. | O | O | O | O | O |
|  | _______ is good at reading and understanding written maths problems. | O | O | O | O | O |
|  | _______ is extremely talented in logical mathematical thinking. | O | O | O | O | O |
|  |  | Strongly Disagree | Disagree | Uncertain | Agree | Strongly Agree |
|  | When somebody describes their house to him/her, he/she is very good at imagining what it looks like. | O | O | O | O | O |
|  | ‘_______ finds orientation in a foreign city using a map extremely easy. | O | O | O | O | O |
|  | _______ can very easily imagine what an object would look like from a different point of view. | O | O | O | O | O |
|  | _______ is good at explaining to other people which route to use. | O | O | O | O | O |
|  | _______ is good at finding his/her way in an unknown area. | O | O | O | O | O |
|  | Estimating the size of a room is easy for _______. | O | O | O | O | O |
|  | _______ can easily imagine three-dimensional objects. | O | O | O | O | O |
|  | ‘______ finds it easy to judge the distance between two points/locations. | O | O | O | O | O |
|  | _______ is extremely talented at spatial thinking. | O | O | O | O | O |

Peer-Estimate Questionnaire 2

In the following section you will find a number of statements. Please answer honestly in the way that you think best describes _________’s opinion. Please answer this survey in a timely manner and bear in mind that it is only about ________’s personal opinion. Please only mark one option per statement. There are no right or wrong answers to this.

|  |  | Strongly Disagree | Disagree | Uncertain | Agree | Strongly Agree |
| --- | --- | --- | --- | --- | --- | --- |
|  | _______ is good at finding original headlines for drawings or writings. | O | O | O | O | O |
|  | _______ constantly has new ideas. | O | O | O | O | O |
|  | In his/her spare time _______ likes to be active in an artistic way. | O | O | O | O | O |
|  | _______ has the skill to look at a problem from different perspectives. | O | O | O | O | O |
|  | It is very easy for _______to find different possible solutions to a problem. | O | O | O | O | O |
|  | _______ has quite unconventional and flamboyant ideas. | O | O | O | O | O |
|  | _______ is very curious and interested in the world. | O | O | O | O | O |
|  | _______ can find many different uses for everyday objects. | O | O | O | O | O |
|  | _______ is extremely talented at creative tasks. | O | O | O | O | O |

Peer-Estimate Questionnaire 3

In the following section you will find a number of statements. Please answer honestly in the way that you think best describes _________’s opinion. Please answer this survey in a timely manner and bear in mind that it is only about ________’s personal opinion. Please only mark one option per statement. There are no right or wrong answers to this.

|  |  | Strongly Disagree | Disagree | Uncertain | Agree | Strongly Agree |
| --- | --- | --- | --- | --- | --- | --- |
|  | If something really bothers _______ he/she usually tries to analyse his/her feelings‘ / ‘usually tries to understand why he/she feels like that. | O | O | O | O | O |
|  | If a situation really concerns _______ he/she tries to avoid similar situations in the future. | O | O | O | O | O |
|  | If a topic really aggravates _______ he/she still tries to stay calm in dealing with it. | O | O | O | O | O |
|  | _______ is very good at expressing his/her own feelings in a clear and understandable way. | O | O | O | O | O |
|  | If _______ is feeling down he/she usually knows how to cheer himself/herself up again.’. | O | O | O | O | O |
|  | Even if others tell _______ he/she really did very well at something he/she is still unhappy with it/with the result. | O | O | O | O | O |
|  | _______ is very good at recognizing and differentiating his/her own feelings. | O | O | O | O | O |
|  | If necessary _______ can easily control his/her own feelings. | O | O | O | O | O |
|  | _______ is extremely talented at regulating his/her own feelings. | O | O | O | O | O |

|  |  | Strongly Disagree | Disagree | Uncertain | Agree | Strongly Agree |
| --- | --- | --- | --- | --- | --- | --- |
|  | _______ is very good at helping others feel better when they are feeling down – without simply distracting them from their problem. | O | O | O | O | O |
|  | _______’s feelings tend to make him/her react in an inappropriate way to others. | O | O | O | O | O |
|  | If a friend is sad, _______ tries to support him/her in finding a solution to the problem. | O | O | O | O | O |
|  | If _______ is struggling to find a solution to a friend’s problems, he/she tries to find somebody else who can offer them better help. | O | O | O | O | O |
|  | If _______ hurts somebody’s feelings he/she realizes this and apologizes. | O | O | O | O | O |
|  | _______ is good at empathizing. | O | O | O | O | O |
|  | _______ has lots of ideas for solving interpersonal problems. | O | O | O | O | O |
|  | When interacting with others _______ is very polite. | O | O | O | O | O |
|  | _______ is good at understanding the emotions of others and reacting to them in an appropriate way. | O | O | O | O | O |
|  | _______ is extremely talented at interacting with other people. | O | O | O | O | O |
